# Computational Model of Heterogeneity in Melanoma: Designing Therapies and Predicting Outcomes

**DOI:** 10.3389/fonc.2022.857572

**Published:** 2022-04-14

**Authors:** Arran Hodgkinson, Dumitru Trucu, Matthieu Lacroix, Laurent Le Cam, Ovidiu Radulescu

**Affiliations:** ^1^ Living Systems Institute, University of Exeter, Exeter, United Kingdom; ^2^ Division of Mathematics, University of Dundee, Dundee, United Kingdom; ^3^ IRCM, Institut de Recherche en Cancérologie de Montpellier, INSERM U1194, Univ Montpellier, Institut régional du Cancer de Montpellier, Montpellier, France; ^4^ Equipe Labélisée Ligue contre le cancer, Paris, France; ^5^ LPHI, University of Montpellier and CNRS UMR 5235, Montpellier, France

**Keywords:** cancer heterogeneity, melanoma, targeted treatment, single cell data, mathematical modeling

## Abstract

Cutaneous melanoma is a highly invasive tumor and, despite the development of recent therapies, most patients with advanced metastatic melanoma have a poor clinical outcome. The most frequent mutations in melanoma affect the BRAF oncogene, a protein kinase of the MAPK signaling pathway. Therapies targeting both BRAF and MEK are effective for only 50% of patients and, almost systematically, generate drug resistance. Genetic and non-genetic mechanisms associated with the strong heterogeneity and plasticity of melanoma cells have been suggested to favor drug resistance but are still poorly understood. Recently, we have introduced a novel mathematical formalism allowing the representation of the relation between tumor heterogeneity and drug resistance and proposed several models for the development of resistance of melanoma treated with BRAF/MEK inhibitors. In this paper, we further investigate this relationship by using a new computational model that copes with multiple cell states identified by single cell mRNA sequencing data in melanoma treated with BRAF/MEK inhibitors. We use this model to predict the outcome of different therapeutic strategies. The reference therapy, referred to as “continuous” consists in applying one or several drugs without disruption. In “combination therapy”, several drugs are used sequentially. In “adaptive therapy” drug application is interrupted when the tumor size is below a lower threshold and resumed when the size goes over an upper threshold. We show that, counter-intuitively, the optimal protocol in combination therapy of BRAF/MEK inhibitors with a hypothetical drug targeting cell states that develop later during the tumor response to kinase inhibitors, is to treat first with this hypothetical drug. Also, even though there is little difference in the timing of emergence of the resistance between continuous and adaptive therapies, the spatial distribution of the different melanoma subpopulations is more zonated in the case of adaptive therapy.

## Introduction

More than one half of melanomas carry mutations of the gene coding the BRAF kinase, a key upstream component of the MAPK signaling pathway, which is involved in cell growth and proliferation. In this pathway, BRAF phosphorylates and activates MEK that in turn phosphorylates and activates ERK, a potent effector that induces the transcription of many important genes that play a dominant role in survival and development of tumor cells. In melanoma, targeted therapies based on BRAF inhibitors (vemurafenib, dabrafenib, encorafenib) and MEK inhibitors (trametinib, cobimetinib, binimetinib) aim at reducing the activity of this key signaling cascade (1–4). BRAF inhibitors act differentially on cancer and healthy cells. Indeed, elevated MEK and ERK activity is induced mainly by BRAF dimers, and less by monomers. In BRAF-mutated melanomas, RAS-GTP levels are insufficient to promote BRAF dimerization, therefore the inhibition of BRAF monomers is sufficient for ERK inactivation. This specificity reduces the toxicity of this type of treatment (5). Although the treatment based on these kinase inhibitors initially leads to efficient tumor regression, resistance appears almost systematically. Several mechanisms have been associated to acquired resistance, such as RAS mutation, receptor tyrosine kinase activation that either compromise ERK inactivation or induce other survival pathways such as PI3K/AKT (5).

We focus here on a non-exclusive, but different cause of resistance, that involves the development of several drug tolerant cell states by non-genetic mechanisms. The non-genetic nature of adaptive resistance in melanoma was first suggested by the reversibility of this process: resistant tumors can re-sensitize upon a drug holiday (6, 7). Coexistence of sensitive and resistant cells with anti-correlated fitness in treated and untreated conditions can also explain apparent tumor re-sensitization in the absence of drug by positive selection of sensitive cells and negative selection of resistant cells, without the need for transitions between different cell states (8). Moreover, single cell RNA analysis has demonstrated plastic transitions between distinct cellular phenotypes in cell lines (9–11) and in patient-derived xenograft (PDX) mouse models (12, 13) submitted to BRAF/MEK inhibitors. Treatment-induced transitions between cell states have robust features, common to many patient-derived cultures and different cell lines (11). Between the melanocytic and mesenchymal-like states which represent the sensitive and resistant extremes there are intermediate states resembling nutrient-starved cells and evolving *via* several trajectories towards mensenchymal-like states. The intermediate states and the trajectories originating therein show intrinsic variability of the gene expression, which suggests that the transitions between states are continuous rather than discrete (9, 11, 12).

These fundamental findings could be used to design new therapeutic strategies to avoid resistance. The re-sensitization, either real or apparent, arising when resistant cells are slowly growing in untreated conditions, suggest that a discontinuous adaptive treatment, alternating ‘on’ and ‘off’ drug periods, may be able to control tumor size, at least for some time. Combination therapy may also depend on one’s capacity to predict the changes induced by the primary tumor treatment, in space and time. For instance, drug tolerant neural crest stem cells, which are enriched upon treatment with BRAF/MEK inhibitors, display an RXR-driven signature, suggesting that these cells could eventually be targeted pharmacologically by using RXR-inhibitors (12). Besides anti-BRAF/MEK targeted therapies, the recent discovery that immune checkpoint inhibitors, targeting regulatory molecules on T lymphocytes (anti-CTLA4, anti-PD-1, and anti-PD-L1), are highly efficient in melanoma patients has revolutionized the treatment of metastatic melanoma. However, each treatment modality has limitations. While treatment with targeted therapies is associated with a strong beneficial short-term response but is followed by systematic resistance, treatment with immune checkpoint inhibitors has a lower response rate but associates with better long-term responses on a subset of melanoma patients. Thus, despite these considerable improvements in melanoma treatment, the development of new clinical strategies remains necessary and a better understanding of melanoma biology is likely to provide additional therapeutic options to patients with resistant cancers (14, 15).

In this paper we use a computational framework to study the heterogeneity of melanoma and develop a predictive model for various therapeutic outcomes. We base our model on data obtained in MEL06 patient-derived melanoma cells, which were demonstrated to develop non-genetic resistance to BRAF/MEK inhibitors (12). Given the complexity of the resistance mechanisms in melanoma, our conclusions may not hold true for all melanomas, which may evolve during treatments through multiple mechanisms of resistance.

## Results

### Multidimensional, Data Driven Model of Heterogeneity

**Figure 1:**
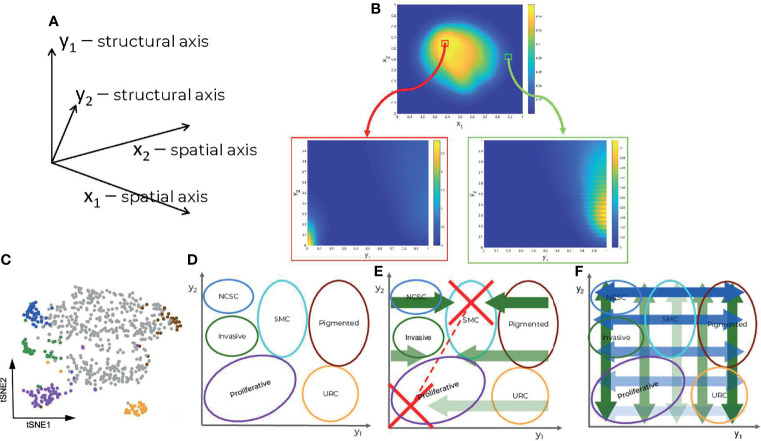
Components of the data driven heterogeneity model: **(A)** Dimensions of the model. **(B)** Multidimensional cell distributions predicted by the model emphasizing treatment induced zoning. **(C)** Reduced representation of single cell expression data, from (12). **(D)** Cell states represented as domains in structural/gene expression space. **(E)** Directed (advective) structural fluxes. **(F)** Undirected (diffusive) structural fluxes.

Our main assumption is that under treatment melanoma cells undergo a series of non-genetic transitions, leading to drug tolerant and resistant cell states. Contrary to more traditional models of heterogeneity that consider a finite number of discrete cell states (16), our model can cope with a continuous spectrum of states. In this model, cell populations are represented as distributions (heatmaps) over many dimensions; spatial, coping with cell motility and cell interactions with extracellular matrix, diffusive drug and signaling molecules, but also structural, representing internal cell-state variables such as gene expression, signaling, and metabolic activities (**Figures 1A, B**). An interesting possibility is to use single cell data and feature extraction methods such as t-distributed stochastic neighbor embedding (t-SNE) in order to define reduced structural dimensions (**Figures 1C, D**). In this case, the distributions (heatmaps) predicted by the model (**Figure 1B**) can be directly compared to the empirical single cell distributions. We call this approach ‘mesoscopic’ as it is intermediate between a microscopic approach, which simulates each cell individually, and a macroscopic one, in which the cell-to-cell variability is averaged out. Even though this method can be applied to any type of single cell data (transcriptomic, proteomic or metabolomic), our analysis is based on the single cell mRNA sequencing data from (12).

In order to clarify the structural dimensions of our tumor, we distinguished six different melanoma subpopulations; namely proliferative, invasive, pigmented cells, neural-crest stem cells (NCSCs), starved-like melanoma cells (SMCs), and uncharacterized resistant cells (URCs). These are schematically represented in the structural plane in **Figure 1D** and are in line with previous experimental results (12). It should be recalled that individual cells may concurrently occupy several states, existing in a continuum of gene expression profiles across the structural domain.

The model predicts dynamic heterogeneity, meaning that the multidimensional distributions depend also on time. The evolution of these distributions is driven by spatial fluxes, involving undirected (diffusive) and directed (advective) spatial cell motility, and by structural fluxes, corresponding to changes of the cell state. The undirected spatial diffusion fluxes describe a cellular spatial random walk process, whereas directed spatial fluxes describe controlled cell migration mediated by adhesive extracellular matrix substrates or to sites of more elevated nutritional content (see *Methods* and **Supplementary Methods**). The undirected structural fluxes (structural diffusion) correspond to random changes of the cell state leading to the spread of the cell distributions (increased heterogeneity) without changes of modal positions in the structural dimensions. The directed structural fluxes (structural advection) correspond to deterministic changes of the cell state, leading to shifts of the distribution modes. The cell distribution dynamics, represented as one 4D (2 spatial and 2 structural dimensions) partial differential equation (PDE), is coupled to five other 2D (2 spatial dimensions) PDEs coping with the spatial distribution of other variables such as extracellular nutritional environment (ECNE), chemo-attractant molecules (surrogate for mediated cell-cell communications), and drug concentrations. The effect of the drugs on the cells’ distribution is taken into account in the negative (degradative) source terms that depend on their position within the structural domain, i.e., on the cell state. For details, see *Methods*.

### Targeted Treatment Exacerbates Heterogeneity

The model recapitulates the dynamics of the cell heterogeneity observed in (12) (see **Movies S1**, **S2**). Starting with a naïve tumor containing a population of sensitive melanocytes, several cell subpopulations are induced by the therapy. In our simulations, this is seen by the multimodality of the cell population’s structural distribution, with positions of the modes depending on time. As shown in **Figure 1D**, each sub-population is characterized by the position of the mode and by its spread in the structural domain. For a more quantitative approach, we use the variance in the cell structural distribution as a metric and show that heterogeneity increases with time upon drug administration (**Figure S1**).

The model predicts the typical three phase tumor growth curve under kinase inhibitors; a first phase wherein the tumor responds and shrinks, a second phase wherein the tumor is no longer visible corresponding to the minimal residual disease (MRD), and a third phase during which tumor growth resumes after the emergence of resistance. During the MRD phase, heterogeneity strongly increases through continuous spreading of the cell distributions in the structural dimensions and, moreover, by development of co-existing, drug-tolerant, intermediate states between sensitivity and resistance (multi-modality, see **Movie S2**).

### Order in Combination Therapy Matters

We have tested, in our computational setting, combination therapies by successively applying two differing types of treatments: (1) using BRAF/MEK inhibitors (BRAF/MEKi) as in (12), and (2) a hypothetical cancer treatment (HCT). We have considered that the tumor has the same intrinsic dynamics, defined by the same diffusion and advection terms, for the two treatments. In particular, the cell states and their transitions will be the same for the two treatments. However, the two treatments eliminate cells differently, depending on their states. This difference between treatments was modeled by using a drug response function, defining how the drug dependent cell degradation changes with the cell state. This function peaks in the modal position of the primary tumor, in the case of BRAF/MEKi, or in the positions of the BRAF/MEKi resistant states, typically invasive and URC cell populations, in the case of HCT (see *Methods* and **Figure S2**). Applied alone, the BRAF/MEKi treatment induces immediate and drastic tumor reduction, followed by MRD and development of resistance after approximately four months of continuous administration of the drugs. The HCT treatment leads to a mild response initially, but like BRAF/MEKi treatment, induces tumor adaptation. However, the representation of invasive and URC cell states is only moderate because they are now more effectively eliminated.

**Figure 2:**
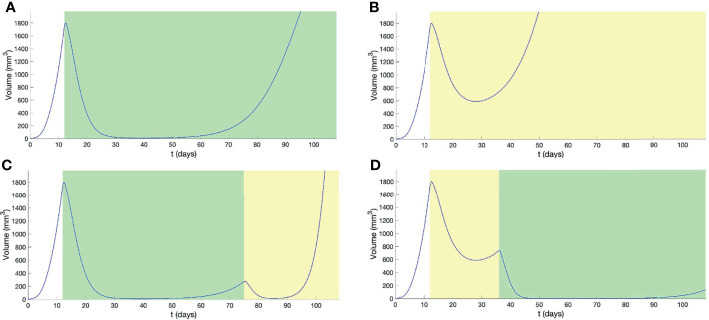
Outcomes from combination therapy, with treatment intervals indicated by graphical shading for BRAF/MEKi (*green*) and HCT (*yellow*). Panels show the outcomes from **(A)** continuous BRAF/MEKi, **(B)** continuous HCT, **(C)** combination BRAF/MEKi then HCT, and **(D)** combination HCT then BRAF/MEKi treatment regimes.

Treatments using BRAF/MEKi (**Figure 2A** and **Movie S3**) or HCT (**Figure 2B** and **Movie S4**), alone, resulted in a re-establishment of initial tumor volume, prior to the end of the study period, with HCT inducing resistance far earlier than BRAF/MEKi. For the combination therapy, BRAF/MEKi then HCT, we observe a later time-point for the re-establishment of the initial tumor volume, in comparison to BRAF/MEKi only, but still resulted in a significant increased tumor growth rate (**Figure 2C** and **Movie S5**). Starting first with HCT and then using BRAF/MEKi, however, was a better strategy that significantly delayed resistance and also reduced the tumor load by combining the advantages of the two treatments (**Figure 2D** and **Movie S6**).

### Output in Terms of Heterogeneity Depends on the Therapeutic Strategy

**Figure 3:**
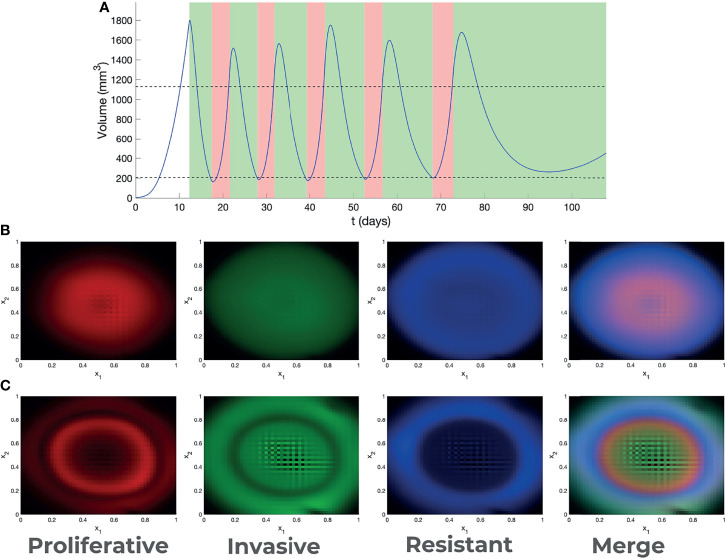
Adaptive therapy. **(A)** Outcome of adaptive therapy, with BRAF/MEKi treatment intervals indicated in *green* and drug hollidays in *pink*. Decision about treatment is taken every day. Treatment is applied if the volume is higher than the upper threshold, stopped if the volume is lower than the lower threshold (thresholds are indicated as dotted lines). Spatial heterogeneity (zoning) generated by continuous **(B)** and adaptive **(C)** therapy.

The dynamics of melanoma cells submitted to kinase inhibitors is typically robust. In the case of adaptive therapy, although the intermediate dynamics is modified by allowing the tumor to grow before re-applying treatment, our model predicted that resistance development cannot be avoided (**Movies S7**, **S8**). However, in terms of spatial heterogeneity, the outcome is much more variable. In **Figure 3** we have represented the spatial distribution of different cell states at the end of MRD and beginning of resistance, for various treatments. In all cases cell states depend on position, a phenomenon called zoning. The details of this phenomena depend on the type of therapy. Our model predicted that the adaptive therapy generates more pronounced zoning, with steeper and mutually exclusive patterns (**Figure 3**) than those predicted under continuous therapies.

## Discussion and Conclusion

Treatment by kinase inhibitors leads to a heterogeneity upsurge in melanoma. At least part of this heterogeneity is generated by non-genetic mechanisms and involves continuous modifications of gene expression programs which lead to transitions between cell states. Our mathematical model captures the essential features of non-genetic transitions and explains the heterogeneous dynamics by diffusive and advective spatial and structural fluxes. The increased heterogeneity results from the multiplicity of drug tolerant and resistant states induced by the treatment, and from cooperative strategies in a spatially heterogeneous tumor where resistant cells protect sensitive cells from elevated drug exposure.

Moreover, our model predicts *in silico* the outcomes of various therapies.

Here, we show that, considering combination therapies, it is better to treat first with a less effective hypothetical drug, targeting sub-populations that develop during tumor resistance phases, before treating with BRAF/MEKi. The explanation of this rather counter-intuitive result can be found in the cell population dynamics. We supposed that the intermediate response of the tumor to any of the treatments results in increased heterogeneity by non-genetic processes. If the first applied treatment is BRAF/MEKi, this acts mainly on cells belonging to early modes and there is a non-negligible probability that some cells escape treatment and become resistant. The same probability is very small when the first applied treatment is HCT that acts preferentially on cells belonging to late modes; the role of HCT initial application is to avoid starting the BRAF/MEKi treatment with some cells that are not sensitive. Then, the application of BRAF/MEKi kills practically all the remaining cells and resistance takes much longer time to develop (see **Movies S5**, **S6**). One should note that, due to structural diffusion, any cell state can, in theory, give rise to all other cell states. Therefore, in order to confine cells to BRAF/MEKi-sensitive modes, the drug has to act on a large domain of cell states, not only on a single intermediate drug tolerant sub-population. This is difficult to perform using targeted therapies.

A possible candidate for the hypothetical cancer treatment (HCT) is the drug family of immune checkpoint inhibitors (ICIs). Although this treatment does not act directly on melanoma cells, it can have a differential indirect effect on melanoma sub-populations, and acts more generally than targeted treatments. Very recent Phase III trials combining kinase inhibitors and ICIs show that starting a first line treatment with ICIs leads to better results in terms of survival time and duration of response than starting with kinase inhibitors (17). This is explained if checkpoint inhibitors induce effective prior elimination of resistant stage sub-populations. There are, however, other interpretations of the interplay between kinase inhibitors and immunotherapy. Obenauf et al. (18) showed that kinase inhibitors induce changes in the stroma and cell secretome and hypothesized changes of immune cells infiltration. Other authors suggested that treatment with BRAFi leads to favorable changes in the tumor microenvironment in synergy with immune checkpoint inhibitors (see (14) for a review). The interactions between the immune cells and the various melanoma sub-populations are still poorly defined. We hope that future experimental and modelling work in the field, will elucidate the mechanistic aspects of these interactions.

Simple models of adaptive therapy were based on the idea that, in the absence of drugs, resistant cells grow more slowly than sensitive cells (10). It is believed that this fitness advantage allows sensitive cells to recover at least partially during a drug holiday. Although this effect is present in our model, it is compensated by structural and spatial diffusion that led to increased heterogeneity and delay only moderately the time to resistance. The resulting tumor, however, depends on the type, continuous or adaptive, of treatment. Irrespective of the treatment, zoning is a population-scale strategy to increase the mean fitness by cooperative protection of sensitive cells by resistant cells. In the adaptive treatment, the growth of sensitive cells is also favored by drug holidays, which lead to a more pronounced zoning.

From a theoretical perspective, our model shows the interplay between directed and undirected structural fluxes for the development of plasticity and heterogeneity. Undirected fluxes correspond to diffusion and random changes of cell states. As well known in physics, or in neutral theory of molecular evolution, free diffusion can reach any state from any other state if one waits a time proportional to the square of the state change. In the presence of treatment, diffusion is not free and has to cross barriers generated by the drugs action. In this case, the escape transition time is exponential. The escape transition time and the proportion of escaping cells depend on the position, height, and width of the barriers, which are different for different treatments. This dependence further explains why order matters in combination therapy and why heterogeneity may differ when employing adaptive strategies, since the barrier is time-transient. Another important theoretical aspect is the symmetry breaking induced by the treatment. Although a barrier can be crossed in both directions, the transition probability is asymmetric if one of the states is more stable than the others. This leads to the notion of metastable states hierarchy, in which states are distinguished by the time that cells spend in each one of these; this time can be very long for highly stable states. Adaptive therapies favor the stabilization of one metastable state by alternating treatment and holiday periods. The success of this strategy depends on conditions that may be difficult to guarantee, especially in a multidimensional context and for a spatially heterogeneous drug distribution.

We should nevertheless emphasize that our model is mostly phenomenological with structural dimensions representing nonlinear functions of the gene expression data. As several findings point towards the role of BRAFi in metabolic remodeling (19), it would be very useful to interpret the structure variables in terms of metabolic changes. This is possible within our formalism as metabolic ODE models [see for instance (20)] are transposable into structural advection fluxes, where metabolic stochasticity or uncertainty would translate to diffusive fluxes. This possibility will be investigated in future work. Furthermore, the distribution of blood vessels that are sources of nutrition and drug compounds is an important variable for understanding zoning aspects of cancer adaptation to treatment [see also (21)]. Like in (21), we expect that the spatial distribution of sensitive and resistant cells depends on the distance to these sources. Blood vessels distribution can be reconstructed from *ex vivo* tumor sections (22) and we will use these distributions to increase the realism of future models.

## Methods

### General Formalism

Mesoscale models of cancer heterogeneity are based on partial differential equations and can be generically obtained from the Liouville continuity equation (8, 23, 24). Let us consider that there are *n* types of cells. In this model cells are distinguished by two types of variables, a discrete one representing the type *i* ε {1,…, *n*} and a continuous one ***y*** = (*y*_1_, …, *y_m_*) representing the internal state (vector of concentrations of biochemical species, for instance). Then ***c*** = (*c*_1_,…, *c_n_*) represents a vector of cell distributions satisfying the equation


(1) $$\frac{\partial c\left(x,y,t\right)}{\partial t}=-\nabla_{x}\cdot F_{x}\left(x,y,t\right)-\nabla_{y}\cdot F_{y}\left(x,y,t\right)+S\left(x,y,t\right),$$

where ***x*** is the spatial position, ***y*** is the cell’s internal state (structure variable), ***F_x_*** is the spatial flux, ***F_y_*** is the structural flux and ***S*** is the source term. If the cell’s internal state follows ODEs $\frac{dy}{dt}=\Phi \left(y\right)$ , then the structural flux is advective ***F_y_*** = ***c*** Φ. If the cell’s state follows random Brownian motion in the structural space, then the structural flux is diffusive resulting from Fick’s law ***F_y_*** = –***D*** *_y_* ***∇_y_c***, where ***D_y_*** is the structural diffusion coefficient matrix. The spatial flux contains terms related to cell motility: undirected (diffusion), or directed (chemotaxis, haptotaxis) (25). The source term can integrate cell proliferation, death, and discrete stochastic changes (finite jumps) of the cell state ***y***, other than those included in the continuous flux ***F_y_***.

### Model Derived From Single Cell Expression Data

In this section we present only the broad lines of the model construction. The details can be found in the **Supplementary Methods**.

#### Model Components

Our melanoma progression model has two main components: the cancer cell population density *c*(*t*, *x*, *y*) and the extra-cellular nutritional environment (ECNE) density *v*(*t*, *x*). In our minimal melanoma model, space positions *x*, and structural positions *y* are two dimensional (in space we consider a 2D tumor section, and in structure we use a 2D t-SNE representation of the tumor transcriptome). We also consider spatial gradients of three types of diffusible molecules, namely 1) the nutritional molecular species, provided by the ECNE and consumed by cells, 2) the acidic molecular species, produced by cells and degrading the ECNE as in (26), and 3) drugs.

The fluxes defining the model dynamics have been derived using the following assumptions:

#### Spatial Variables and Fluxes

We assume, consistently with previous mathematical studies of spatial cancer dynamics (25), that the spatial dynamics of melanoma cells are governed by both random (diffusive) and deterministic (advective) components. The random (diffusive) component is assumed to occur as a result of tissue-scale reorientation and volume-filling processes. The deterministic (advective) component is assumed to result from directed cell motility and is driven by cell-environment interactions. In particular, we assume that cells exhibit controlled migration to sites of chemically elevated nutritional content (chemotaxis), as well as to sites of higher ECNE density (haptotaxis).

#### Structural Variables and Fluxes

The definition of the structural variables follows from the data analysis in (12). Unsupervised clustering of single cell mRNA-seq data identified several types of cell sub-populations with distinct transcription states. The high-dimensional transcriptome was compressed to a 2D map using t-distributed stochastic neighbor embedding (t-SNE). The support of this 2D map is our structure space domain. The different transcription states represent sub-domains in this representation (see **Figure 1D**). The cell-state transitions observed experimentally can be represented as diffusion and advective flow in this domain. The flow changes the positions of the cells in the 2D structure domain, moving them from one state to another. Thus, rather than considering a number of distinct cell types, we have built a model with only one cell type whose state can change continuously by the structural fluxes. A cell is added to a sub-population if its state enters the corresponding structural sub-domain and is subtracted if it dies or if it leaves the sub-domain. In order to define the structural fluxes, we start by identifying the sub-domains corresponding to different sub-populations inside the tumor at different times. Although seven transcriptional signatures were identified [**Table S1** of (12)], we focus upon the description of six primary states important for resistance. For their localization in the structural domain, we use cardinal points, as follows:

- Melanoma cells with a “proliferative” signature are predominant in naive tumors, localized south-west (SW).
- Invasive cells are also present in naive tumors. They are localized east (E).
- Pigmented cells expressing markers of differentiation are induced by the treatment. They are localized north-west (NW).
- Neural crest stem cells (NCSC) are enriched by the treatment, have a maximum during the minimal residual disease and are diluted out during the development of resistance. They are localized north-east (NE).
- Starved-like melanoma cells (SMC) are rapidly induced by the treatment, and become predominant during MRD. They are localized north (N).
- Uncharacterized resistant cells (URCs) were not thoroughly biologically investigated, though the model predicts they may have a biological interest. They are localized south-west (SW).

The structural fluxes describe the metabolic adaptation within the structural domain and diffusion-like exchanges between cell populations (**Figures 1E, F**). In order to define these fluxes, we use the following dynamical assumptions:

- Horizontal advection is assumed to stabilize the proliferative (SW) state, since there is no known emergence of URCs prior to resistance;
- SMC states (N) are also stabilized by horizontal advection fluxes that converge towards this state, allowing cells to populate this minimally mitotic state;
- advection is assumed to interpolate linearly at intermediate phenotypes between proliferative cells and SMCs;
- horizontal diffusion is assumed to be maximal in the northern regions of the structural plane and decreases in southern regions, illustrating rare, stochastic transitions between proliferative and URC states;
- vertical diffusion is maximal towards the western and eastern regions, allowing transitions between proliferative, invasive, and NCSC or pigmented and URC populations, but lower transition rates between SMC and southern states.

In principle, by diffusion any cell state can give rise to all cell states. However, advection maintains a certain degree of cellular hierarchy. These assumptions have been made upon a reasoned analysis of the figures presented in (12), as a minimal set of functional assumptions to reproduce observed patterns, but do not necessarily represent an optimal or biologically motivated set of assumptions.

#### Source Terms and Degradation

The source and degradation terms describe cell proliferation and death, respectively. Like in (12) we consider that proliferation is significantly reduced among SMC cells and increased among proliferative cells. We consider that treatment is the only cause of active cell death. Due to the nature of our modelling framework, drugs may target cells with a spectrum of specific expression markers as would be the case in the clinical scenario. In this case, we assume the existence of two particular treatments. Firstly, BRAF and MEK inhibitors were employed within the study conducted by (12) and, as such, are assumed to primarily target a distribution centered around the proliferative population, stretching into the invasive population but with diminished success among cells in the NW of the structural domain (**Figure S2**). Secondly, a hypothetical cancer therapeutic (HCT) has been used for the sake of illustration and targets primarily the invasive and URC cell populations, with an expansive effectiveness span E and SW (**Figure S2**).

#### Spatial Dynamics of Other Components

Given the complexity of the dynamics in the primary cancer cell population, the dynamics of other components have been kept as simple as possible. It is assumed that the ECNE exhibits only a natural restorative growth process, as well as acidic species-induced and natural degradation kinetics. Nutritional and acidic species exhibit diffusion, as well as controlled production, and natural degradation. Finally, the drug species also exhibit diffusion, time dependent administration, as well as natural and cell-based degradation.

## Data Availability Statement

The datasets presented in this study can be found in online repositories. The data can be found here: https://github.com/oradules/Melanoma2D_2021/.

## Author Contributions

AH and OR conceived the project. OR wrote the paper with help from AH, ML and LC. AH designed the model, numerical scheme, and performed the simulations. DT contributed to the general formalism and numerical scheme. All authors contributed to the article and approved the submitted version.

## Funding

We acknowledge financial support from Itmo Cancer on funds administered by INSERM (project MALMO) and from I-SITE MUSE (project MEL-ECO). AH has been funded by the Wellcome Trust Institutional Strategic Support Fund. ML and LLC are financially supported by the Ligue Nationale Contre le Cancer, the Institut National du Cancer (INCa) and the cancéropôle Grand Sud Ouest.

## Conflict of Interest

The authors declare that the research was conducted in the absence of any commercial or financial relationships that could be construed as a potential conflict of interest.

## Publisher’s Note

All claims expressed in this article are solely those of the authors and do not necessarily represent those of their affiliated organizations, or those of the publisher, the editors and the reviewers. Any product that may be evaluated in this article, or claim that may be made by its manufacturer, is not guaranteed or endorsed by the publisher.

## References

[1] Gross S Rahal R Stransky N Lengauer C Hoeflich KP. Targeting Cancer With Kinase Inhibitors. J Clin Invest (2015) 125:1780–9. doi: 10.1172/JCI76094 PMC4463189 25932675

[2] Zhang C Spevak W Zhang Y Burton EA Ma Y Habets G. RAF Inhibitors That Evade Paradoxical MAPK Pathway Activation. Nature (2015) 526:583–6. doi: 10.1038/nature14982 26466569

[3] McClure E Patel A Carr MJ Sun J Zager JS. The Combination of Encorafenib and Binimetinib for the Treatment of Patients With BRAF-Mutated Advanced, Unresectable, or Metastatic Melanoma: An Update. Expert Rev Precis Med Drug Dev (2021) 6:19–29. doi: 10.1080/23808993.2021.1847639

[4] Zhang C Bollag G. Triple Therapy to Outwit the Braf Oncogene. Cancer Discovery (2021) 11:1620–2. doi: 10.1158/2159-8290.CD-21-0378 34284995

[5] Poulikakos PI Rosen N. Mutant BRAF Melanomas–Dependence and Resistance. Cancer Cell (2011) 19:11–5. doi: 10.1016/j.ccr.2011.01.008 21251612

[6] Das Thakur M Salangsang F Landman AS Sellers WR Pryer NK Levesque MP. Modelling Vemurafenib Resistance in Melanoma Reveals a Strategy to Forestall Drug Resistance. Nature (2013) 494:251–5. doi: 10.1038/nature11814 PMC3930354 23302800

[7] Sun C Wang L Huang S Heynen GJ Prahallad A Robert C. Reversible and Adaptive Resistance to Braf (V600e) Inhibition in Melanoma. Nature (2014) 508:118–22. doi: 10.1038/nature13121 24670642

[8] Hodgkinson A Le Cam L Trucu D Radulescu O. Spatio-Genetic and Phenotypic Modelling Elucidates Resistance and Re-Sensitisation to Treatment in Heterogeneous Melanoma. J Theor Biol (2019) 466:84–105. doi: 10.1016/j.jtbi.2018.11.037 30503930

[9] Fallahi-Sichani M Becker V Izar B Baker GJ Lin JR Boswell SA. Adaptive Resistance of Melanoma Cells to RAF Inhibition *via* Reversible Induction of a Slowly Dividing De-Differentiated State. Mol Syst Biol (2017) 13:905. doi: 10.15252/msb.20166796 28069687 PMC5248573

[10] Smalley I Kim E Li J Spence P Wyatt CJ Eroglu Z. Leveraging Transcriptional Dynamics to Improve BRAF Inhibitor Responses in Melanoma. EBioMedicine (2019) 48:178–90. doi: 10.1016/j.ebiom.2019.09.023 PMC6838387 31594749

[11] Wouters J Kalender-Atak Z Minnoye L Spanier KI De Waegeneer M González-Blas CB. Robust Gene Expression Programs Underlie Recurrent Cell States and Phenotype Switching in Melanoma. Nat Cell Biol (2020) 22:986–98. doi: 10.1038/s41556-020-0547-3 32753671

[12] Rambow F Rogiers A Marin-Bejar O Aibar S Femel J Dewaele M. Toward Minimal Residual Disease-Directed Therapy in Melanoma. Cell (2018) 174:843–55. doi: 10.1016/j.cell.2018.06.025 30017245

[13] Marine JC Dawson SJ Dawson MA. Non-Genetic Mechanisms of Therapeutic Resistance in Cancer. Nat Rev Cancer (2020) 20:743–56. doi: 10.1038/s41568-020-00302-4 33033407

[14] Naderi-Azad S Sullivan R. The Potential of BRAF-Targeted Therapy Combined With Immunotherapy in Melanoma. Expert Rev Anticancer Ther (2020) 20:131–6. doi: 10.1080/14737140.2020.1724097 PMC7150269 32003263

[15] Ma VT Daignault-Newton S Waninger JJ Journey S Chopra Z Tezel A. The Impact of BRAF Mutation Status on Clinical Outcomes With Anti-PD-1 Monotherapy Versus Combination Ipilimumab/Nivolumab in Treatment-Naïve Advanced Stage Melanoma. Pigment Cell Melanoma Res (2021) 34:629–40. doi: 10.1111/pcmr.12944 33128316

[16] Delitala M Lorenzi T. A Mathematical Model for Progression and Heterogeneity in Colorectal Cancer Dynamics. Theor Population Biol (2011) 79:130–8. doi: 10.1016/j.tpb.2011.01.001 21238471

[17] Atkins MB Lee SJ Chmielowski B Ribas A Tarhini AA Truong TG. Dreamseq (Doublet, Randomized Evaluation in Advanced Melanoma Sequencing): A Phase III Trial–ECOG-ACRIN Ea6134. *J Clin Onco* (2021)39(36_suppl):356154. doi: 10.1200/JCO.2021.39.36_suppl.356154

[18] Obenauf AC Zou Y Ji AL Vanharanta S Shu W Shi H. Therapy-Induced Tumor Secretomes Promote Resistance and Tumor Progression. Nature (2015) 520:368–72. doi: 10.1038/nature14336 PMC4507807 25807485

[19] Corazao-Rozas P Guerreschi P Jendoubi M André F Jonneaux A Scalbert C. Mitochondrial Oxidative Stress Is the Achille’s Heel of Melanoma Cells Resistant to BRAF-Mutant Inhibitor. Oncotarget (2013) 4:1986. doi: 10.18632/oncotarget.1420 24161908 PMC3875764

[20] Jia D Lu M Jung KH Park JH Yu L Onuchic JN. Elucidating Cancer Metabolic Plasticity by Coupling Gene Regulation With Metabolic Pathways. Proc Natl Acad Sci (2019) 116:3909–18. doi: 10.1073/pnas.1816391116 PMC6397570 30733294

[21] Kumar S Sharife H Kreisel T Mogilevsky M B+ar-Lev L Grunewald M. Intra-Tumoral Metabolic Zonation and Resultant Phenotypic Diversification Are Dictated by Blood Vessel Proximity. Cell Metab (2019) 30:201–11. doi: 10.1016/j.cmet.2019.04.003 31056286

[22] Kiemen A Braxton AM Grahn MP Han KS Babu JM Reichel R. *In Situ* Characterization of the 3d Microanatomy of the Pancreas and Pancreatic Cancer at Single Cell Resolution. bioRxiv (2020). doi: 10.1101/2020.12.08.416909

[23] Hodgkinson A Chaplain MAJ Domschke P Trucu D. Computational Approaches and Analysis for a Spatio-Structural-Temporal Invasive Carcinoma Model. Bull Math Biol (2018) 80:701–37. doi: 10.1007/s11538-018-0396-4 29500719

[24] Hodgkinson A Radulescu O Uzé G Trucu D. Signal Propagation in Sensing and Reciprocating Cellular Systems With Spatial and Structural Heterogeneity. Bull Math Biol (2018) 80:1900–36. doi: 10.1007/s11538-018-0439-x 29721746

[25] Chaplain MAJ Lolas G. Mathematical Modelling of Cancer Invasion of Tissue: Dynamic Heterogeneity. Networks Heterog Media (2006) 1:399–439. doi: 10.3934/nhm.2006.1.399

[26] Gatenby RA Gawlinski ET. A Reaction-Diffusion Model of Cancer Invasion. Cancer Res (1996) 56:5745–53. 8971186

