# Supplementary material for: Computational Model of Heterogeneity in Melanoma: Designing Therapies and Predicting Outcomes
Source: Front Oncol. 2022 Apr 14;12:857572. doi: 10.3389/fonc.2022.857572 (PMC9046868; doi:10.3389/fonc.2022.857572)
Supplement: Supplementary file 1 [file Presentation_1.pdf]

# Supplementary Material

## 1 SUPPLEMENTARY METHODS

### Description of the Mathematical Model

*Cancer cell population dynamics.* Using a novel framework (given in eq. (1) of the main text) for higher-dimensional mathematical modelling of population dynamics in cancer Hodgkinson et al. (2019), we are here able to represent the dynamics of the population in time, space, and approximated gene expression levels. The approach results in partial differential equations in a number of dimensions equal to the spatial dimension plus the gene expression dimensions. The latter are reduced to 2 through the use of t-distributed stochastic neighbourhood embedding (t-SNE) method on single cell transcriptomics data Rambow et al. (2018).

The cell population is represented by a density function $c(\boldsymbol{x}, \boldsymbol{y})$ defined on spatial and structural variables $(\boldsymbol{x}, \boldsymbol{y})$, where $\boldsymbol{x}, \boldsymbol{y} \in [0, 1]^2$. The spatial density is the marginal density $c_{\boldsymbol{x}}(\boldsymbol{x}) = \int c(\boldsymbol{x}, \boldsymbol{y}) d\boldsymbol{y}$.

Cell population evolves as a result of spatial fluxes, structural fluxes, and cell sources (proliferation and degradation).

Spatial fluxes describe the movement of cells in space. As in previous models of the spatial evolution of cancer Chaplain and Lolas (2005); Hodgkinson et al. (2019), our model considers that cells may move either in a disorganised, or in an directed manner. The disorganised movement corresponds to the space diffusion flux that, according to the Fick law, is proportional to the gradient of cell density and is directed opposite to this gradient. Directed movement corresponds to chemotactic, or haptotactic fluxes that are proportional to chemical gradients, or to the gradient of ECNE, respectively. In both cases cells move uphill gradients, towards higher densities of cells in chemotaxis, or towards higher density of ECNE in haptotaxis. We consider two types of chemotactic gradients: $m_1(\boldsymbol{x})$ is a nutrient produced by ECNE and $m_2(\boldsymbol{x})$ a chemo-attractant produced by the cancer cells.

The spatial flux term is, therefore, given by:

$$\boldsymbol{F}_{\boldsymbol{x}}(\boldsymbol{x}, \boldsymbol{y}, t) = c\left(1 - \int_{\mathbb{P}} c\, d\boldsymbol{y}\right)\Big(\chi_1 \boldsymbol{\nabla}_{\boldsymbol{x}} m_1 + \chi_2 \boldsymbol{\nabla}_{\boldsymbol{x}} m_2 + \boldsymbol{\nabla}_{\boldsymbol{x}} v\Big) - D_c \boldsymbol{\nabla}_{\boldsymbol{x}} c\,. \tag{S1}$$

The factor $\left(1 - \int_{\mathbb{P}} c\, d\boldsymbol{y}\right)$ has a stabilizing role and was introduced to avoid the Keller-Siegel instability (Keller and Segel, 1970).

Structural fluxes describe the continuous changes of the cell state described as changes of gene expression. Although these changes can be modeled mechanistically using gene and protein interaction networks, in this paper we use a phenomenological model of structural fluxes, based on single cell transcriptomic data. Structural fluxes are also of two types, diffusive and advective, corresponding to random, zero mean, changes of the cell state and deterministic changes of the cell state, respectively. For the diffusive terms, we assume that these vary with the two structural variables $y_1$ and $y_2$ as described in the Methods section and Figure 1f in the main text. The advective terms are similarly described in Methods and Figure 1e in the main text and are assumed to have the proliferative and

SMC subpopulations, as well as all the points linearly interpolated between these states as stable steady states. The stability of these states was considered higher for larger $y_2$, which was modelled as a linear factor $(1-r_{min})y_2 + r_{min}$, $0 < r_{min} < 1$, in the advection flux. Thus, we have used the following model for structural fluxes:

$$\begin{aligned} F_{\boldsymbol{y}}(\boldsymbol{x},\boldsymbol{y},t) = & \begin{bmatrix} r_\mu \\ 0 \end{bmatrix} \left( y_1 - \frac{\xi_{\phi_{\max}} - \xi_{\phi_{\text{hyp}}}}{\nu_{\phi_{\max}} - \nu_{\phi_{\text{hyp}}}} (y_2 - \nu_{\phi_{\text{hyp}}}) - \xi_{\phi_{\text{hyp}}} \right) \Big( (1 - r_{\min}) y_2 + r_{\min} \Big) c - \\ & - \operatorname{diag} \begin{bmatrix} D_1 y_2 \\ 4 D_2 \left( y_1 - \frac{1}{2} \right)^2 \end{bmatrix} \boldsymbol{\nabla}_{\boldsymbol{y}} c \, , \end{aligned} \tag{S2}$$

where $(\xi_{\phi_{\text{hyp}}}, \nu_{\phi_{\text{hyp}}})$, $(\xi_{\phi_{\max}}, \nu_{\phi_{\max}})$ are the structural space centroids of the SMC and the proliferative cells subpopulations, respectively.

Cell sources terms are of two types: positive proliferation terms, and negative degradation terms.

Degradation terms are proportional to a combination of the drug concentrations $p_1 f_1(\boldsymbol{y}) + p_2 f_2(\boldsymbol{y})$, where $p_1$, $p_2$ are the drug concentrations and $f_1(\boldsymbol{y})$, $f_2(\boldsymbol{y})$ are effectiveness functions of the two drugs (see Supplementary Figure 1). The drug effectiveness functions cope with the fact that cells with different states $\boldsymbol{y}$ are eliminated differently by the two drugs.

The proliferation terms take into account competition on resources (logistic growth) and are proportional to the concentration of nutrient $m_1$ produced by ECNE. The proliferation term also contain a factor depending on the cell state $\boldsymbol{y}$, that mimics the cell metabolic activity derived in Rambow et al. (2018). For modelling this factor, it is assumed that all cells have a basic proliferation rate, $\phi_c \phi_0$; that there is a region (centered in $(\xi_{\phi_{\text{up}}}, \nu_{\phi_{\text{up}}})$, in between the states defined in the main text, and close to the west part of the structural domain) exhibiting elevated proliferative activities and an elevated rate, $\phi_c \phi_{\text{up}}$; and that the cells in the proliferative state exhibit the greatest proliferation rate, $\phi_c \phi_{\max}$. Meanwhile, the SMC state is assumed to exhibit a significantly lower rate than the remainder of the domain and this is achieved by dividing the entire term by 1, plus a Gaussian function centered in $(\xi_{\phi_{\text{hyp}}}, \nu_{\phi_{\text{hyp}}})$. We use the following expression for the source terms:

$$\begin{aligned} S(\boldsymbol{x},\boldsymbol{y},t) = & - \delta_c \mathfrak{f}(\boldsymbol{y}).\boldsymbol{p} c + \Phi_c m_1 (1-c) c \begin{bmatrix} \phi_0 \\ \phi_{\text{up}} \\ \phi_{\max} \end{bmatrix} \cdot \begin{bmatrix} 1 \\ \exp\left( - \begin{bmatrix} w_{\phi_{\text{up}},\xi} \\ w_{\phi_{\text{up}},\nu} \end{bmatrix} \cdot \begin{bmatrix} (y_1 - \xi_{\phi_{\text{up}}})^2 \\ (y_2 - \nu_{\phi_{\text{up}}})^2 \end{bmatrix} \right) \\ \exp\left( - \begin{bmatrix} w_{\phi_{\max}} \\ w_{\phi_{\max}} \end{bmatrix} \cdot \begin{bmatrix} (y_1 - \xi_{\phi_{\max}})^2 \\ (y_2 - \nu_{\phi_{\max}})^2 \end{bmatrix} \right) \end{bmatrix} \cdot \\ & \cdot \left( 1 + \exp\left( - \begin{bmatrix} \alpha_{\text{hyp}} \\ \alpha_{\text{hyp}} \end{bmatrix} \cdot \begin{bmatrix} (y_1 - \xi_{\phi_{\text{hyp}}})^2 \\ (y_2 - \nu_{\phi_{\text{hyp}}})^2 \end{bmatrix} + \alpha_{\text{hyp}} w^2_{\phi_{\text{hyp}}} \right) \right)^{-1} . \end{aligned} \tag{S3}$$

*Spatial dynamics of other components.* As well as the cancer cell population, three types of other species are considered in our model; namely the extra-cellular nutritional environment (ECNE), the chemical species, and the drug species.

As in several previous models of cancer invasion Gatenby and Gawlinski (1996); Chaplain and Lolas (2005); Trucu et al. (2013); Hodgkinson et al. (2019), we assume that the cancer cell population invades the stroma by degrading the ECNE to make space for invading cells. Hence, there is a

chemically-mediated degradation, with the rate vector $\boldsymbol{\delta}_v = \begin{bmatrix} 0 \\ \delta_v \end{bmatrix}$ (only the acidic species $m_2$ degrade ECNE). The degradation of the ECNE is also achieved through a natural decay term, with rate $\delta_{v,0}$. Furthermore, the ECNE grows logistically with a rate $\phi_v$.

The chemical species, themselves, diffuse with a rate vector $\boldsymbol{D}_m$. In particular, the current model considers only two chemical species; the nutritional species $m_1$, produced by the ECNE, and the acidic or degradative species $m_2$, produced by the cancer cells. The production of these species is given by the rate vector $\boldsymbol{\phi}_m$. Logistic factors $m_i(1-m_i)$, $i \in \{1,2\}$ multiply the production terms, in order to keep the chemical species concentrations bounded $0 \le m_i \le 1$ for $i \in \{1,2\}$. Chemical species are assumed to undergo degradation with rates given by the vector $\boldsymbol{\delta}_m$.

We consider two drug species, representing BRAF/MEKi (for simplicity we consider only one species even if two kinase inhibitors are administered) and the hypothetical cancer treatment (HCT), respectively. The drug species exhibit diffusive spatial dynamics, with a rate vector $\boldsymbol{D}_p$. The two drug species, are differentially administered to the patient, on the basis of the required treatment. This differential treatment is mathematically represented by the time-dependent, user-defined, function, $\boldsymbol{\Theta}_p(t)$. Drug species are assumed to be degraded by natural processes, with rates $\boldsymbol{\delta}_p$, and by cellular uptake and degradation, with rates $\boldsymbol{\ell}_p$.

*Full System of Equations.* Combining the above equations with the assumptions for the ECNE, chemical, and drug populations, we may then write the following complete set of PDEs as:

$$
\begin{aligned}
\frac{\partial c}{\partial t} &= \boldsymbol{\nabla}_{\boldsymbol{x}} \cdot \left[ diag[\boldsymbol{D}_c] \boldsymbol{\nabla}_{\boldsymbol{x}} c - c \left( 1 - \int_{\mathbb{P}} c \, d\boldsymbol{y} \right) \Big( \chi_1 \boldsymbol{\nabla}_{\boldsymbol{x}} m_1 + \chi_2 \boldsymbol{\nabla}_{\boldsymbol{x}} m_2 + \boldsymbol{\nabla}_x v \Big) \right] + \\
&+ \boldsymbol{\nabla}_{\boldsymbol{y}} \cdot \mathrm{diag} \begin{bmatrix} D_1 y_2 \\ 4 D_2 \left( y_1 - \frac{1}{2} \right)^2 \end{bmatrix} \boldsymbol{\nabla}_{\boldsymbol{y}} c + \\
&+ r_\mu \frac{\partial}{\partial y_1} \left( y_1 - \frac{\xi_{\phi_{\max}} - \xi_{\phi_{\mathrm{hyp}}}}{\nu_{\phi_{\max}} - \nu_{\phi_{\mathrm{hyp}}}} (y_2 - \nu_{\phi_{\mathrm{hyp}}}) - \xi_{\phi_{\mathrm{hyp}}} \right) \Big( (1 - r_{\min}) y_2 + r_{\min} \Big) c - \\
&- \boldsymbol{\delta}_c . \mathrm{diag}[\mathfrak{f}(\boldsymbol{y})] \boldsymbol{p} c + \varPhi_c m_1 (1-c) c \begin{bmatrix} \phi_0 \\ \phi_{\mathrm{up}} \\ \phi_{\max} \end{bmatrix} \cdot \begin{bmatrix} 1 \\ \exp\left( - \begin{bmatrix} w_{\phi_{\mathrm{up}},\xi} \\ w_{\phi_{\mathrm{up}},\nu} \end{bmatrix} \cdot \begin{bmatrix} (y_1 - \xi_{\phi_{\mathrm{up}}})^2 \\ (y_2 - \nu_{\phi_{\mathrm{up}}})^2 \end{bmatrix} \right) \\ \exp\left( - \begin{bmatrix} w_{\phi_{\max}} \\ w_{\phi_{\max}} \end{bmatrix} \cdot \begin{bmatrix} (y_1 - \xi_{\phi_{\max}})^2 \\ (y_2 - \nu_{\phi_{\max}})^2 \end{bmatrix} \right) \end{bmatrix} \cdot \\
&\qquad \cdot \left( 1 + \exp\left( - \begin{bmatrix} \alpha_{\mathrm{hyp}} \\ \alpha_{\mathrm{hyp}} \end{bmatrix} \cdot \begin{bmatrix} (y_1 - \xi_{\phi_{\mathrm{hyp}}})^2 \\ (y_2 - \nu_{\phi_{\mathrm{hyp}}})^2 \end{bmatrix} + \alpha_{\mathrm{hyp}} w_{\phi_{\mathrm{hyp}}}^2 \right) \right)^{-1} , \\
\frac{\partial v}{\partial t} &= \phi_v (1-v) v - \boldsymbol{\delta}_v \cdot \boldsymbol{m} v - \delta_{v,0} v \, , \\
\frac{\partial \boldsymbol{m}}{\partial t} &= \boldsymbol{\nabla}_x \cdot \mathrm{diag}(\boldsymbol{D}_m) \boldsymbol{\nabla}_x m + \mathrm{diag}\left( \begin{bmatrix} \phi_{m_1} v \\ \phi_{m_2} \int_{\mathbb{P}} c \, d\boldsymbol{y} \end{bmatrix} \right) \cdot \begin{bmatrix} m_1 (1 - m_1) \\ m_2 (1 - m_2) \end{bmatrix} - \mathrm{diag}\left( \begin{bmatrix} \delta_{m_1} \\ \delta_{m_2} \end{bmatrix} \right) \boldsymbol{m} \, , \\
\frac{\partial \boldsymbol{p}}{\partial t} &= \boldsymbol{\nabla}_{\boldsymbol{x}} \cdot \mathrm{diag}(\boldsymbol{D}_p) \boldsymbol{\nabla}_{\boldsymbol{x}} \boldsymbol{p} + \boldsymbol{\Theta}_p(t) - \mathrm{diag}(\boldsymbol{\delta}_p) \boldsymbol{p} - \mathrm{diag}(\boldsymbol{\ell}_p) \boldsymbol{p} \int_{\mathbb{P}} c \, d\boldsymbol{y} \, .
\end{aligned} \tag{S4}
$$

The appropriate biological interpretation and value of the model parameters are provided in the Table S1. The code is accessible at https://github.com/oradules/Melanoma2D_2021/.

| **Parameter** | **Interpretation** | **Value** | **Variable** | **Interpretation** |
|---|---|---|---|---|
| $(D_1, D_2)$ | Structural diffusion coeff. | $(3.3 \cdot 10^{-3}, 6.6 \cdot 10^{-3})$ day$^{-1}$ | $c$ | cancer cell density |
| $\boldsymbol{D}_c$ | Spatial diffusion coeff. | $[3.3 \cdot 10^{-5}, 3.3 \cdot 10^{-5}]^T$ day$^{-1}$ | | |
| $(\chi_m, \chi_v)$ | Chemo- and Hapto-taxis coeff. | $(6.6 \cdot 10^{-4}, 6.6 \cdot 10^{-4})$ day$^{-1}$ | | |
| $\boldsymbol{r}_\mu$ | Structural advection coeff. | $13.3 \cdot 10^{-3}$ day$^{-1}$ | | |
| $r_{min}$ | Vertical structural position | 0.17 | | |
| $\phi_c$ | Basic proliferation rate | 1.66 day$^{-1}$ | | |
| $\phi_0$ | Relative proliferation rate | 0.08 | | |
| $\phi_{up}$ | Relative proliferation rate | 0.45 | | |
| $\phi_{max}$ | Relative proliferation rate | 0.47 | | |
| $(\xi_{\phi_{up}}, \nu_{\phi_{up}})$ | Centre of elevated proliferation | $(0.5, 0.2)$ | | |
| $(\xi_{\phi_{max}}, \nu_{\phi_{max}})$ | Centre of maximum proliferation | $(0.15, 0.15)$ | | |
| $(\xi_{\phi_{hyp}}, \nu_{\phi_{hyp}})$ | Centre of hypometabolism | $(0.5, 0.8)$ | | |
| $(w_{\phi_{up},\xi}, w_{\phi_{up},\nu})$ | Width of elevated proliferation | $(2, 35)$ | | |
| $w_{max}$ | Width of maximum proliferation | 20 | | |
| $(\alpha_{\text{hyp}}, w_{hyp})$ | Width of hypometabolic region | $(100, 0.25)$ | | |
| $\boldsymbol{\delta}_c$ | Drug induced cell death rate | $[1.66, 1.66]^T$ day$^{-1}$ | | |
| $\phi_v$ | ECM regeneration rate | $6.6 \cdot 10^{-2}$ day$^{-1}$ | $v$ | ECNE: extra-cellular |
| $\delta_v$ | $m$ dependent ECNE degradation rate | 0.17 day$^{-1}$ | | nutritional environment |
| $\delta_{v0}$ | Basal ECNE degradation rate | $3.3 \cdot 10^{-2}$ day$^{-1}$ | | |
| $(D_{m_1}, D_{m_2})$ | Chemical diffusion coeff. | $(6.6 \cdot 10^{-4}, 6.6 \cdot 10^{-4})$ day$^{-1}$ | $m_1$ | Nutritional molecules |
| $(\delta_{m_1}, \delta_{m_2})$ | Degradation rates $m_1$,$m_2$ | $(0.66, 6.6 \cdot 10^{-2})$ day$^{-1}$ | $m_2$ | Degrading molecules |
| $(\phi_{m_1}, \phi_{m_2})$ | Production rates $m_1$,$m_2$ | $(0.33, 0.33)$ day$^{-1}$ | | |
| $(D_{p_1}, D_{p_2})$ | Drug diffusion coeff. | $(4 \cdot 10^{-3}, 4 \cdot 10^{-3})$ day$^{-1}$ | $p_1$ | BRAF/MEKi |
| $\boldsymbol{\ell}_p$ | $c$ dependent degradation rate | $(2.16, 2.16)$ day$^{-1}$ | $p_2$ | Hypothetical cancer |
| $(\delta_{p_1}, \delta_{p_2})$ | Basal drug degradation rate | $(1.66 \cdot 10^{-2}, 1.66 \cdot 10^{-2})$ day$^{-1}$ | | treatment (HCT) |

**Table S1.** Model variables and parameters. All quantities are non-dimensionalized, with the exception of time whose units are given in days.

## REFERENCES

Hodgkinson A, Le Cam L, Trucu D, Radulescu O. Spatio-genetic and phenotypic modelling elucidates resistance and re-sensitisation to treatment in heterogeneous melanoma. *Journal of theoretical biology* **466** (2019) 84–105.

Rambow F, Rogiers A, Marin-Bejar O, Aibar S, Femel J, Dewaele M, et al. Toward minimal residual disease-directed therapy in melanoma. *Cell* **174** (2018) 843–855.

Chaplain MAJ, Lolas G. Mathematical modelling of cancer cell invasion of tissue: the role of the urokinase plasminogen activation system. *Mathematical Models and Methods in Applied Sciences* **11** (2005) 1685–1734.

Keller EF, Segel LA. Initiation of slime mold aggregation viewed as an instability. *Journal of theoretical biology* **26** (1970) 399–415.

Gatenby RA, Gawlinski ET. A Reaction-Diffusion Model of Cancer Invasion. *Cancer Res.* **56** (1996) 5745–5753.

Trucu D, Lin P, Chaplain MAJ, Wang Y. A multiscale moving boundary model arising in cancer invasion. *Multiscale Model. Simul.* **11** (2013) 309–335.

# 2 SUPPLEMENTARY FIGURES

## 2.1 Figures

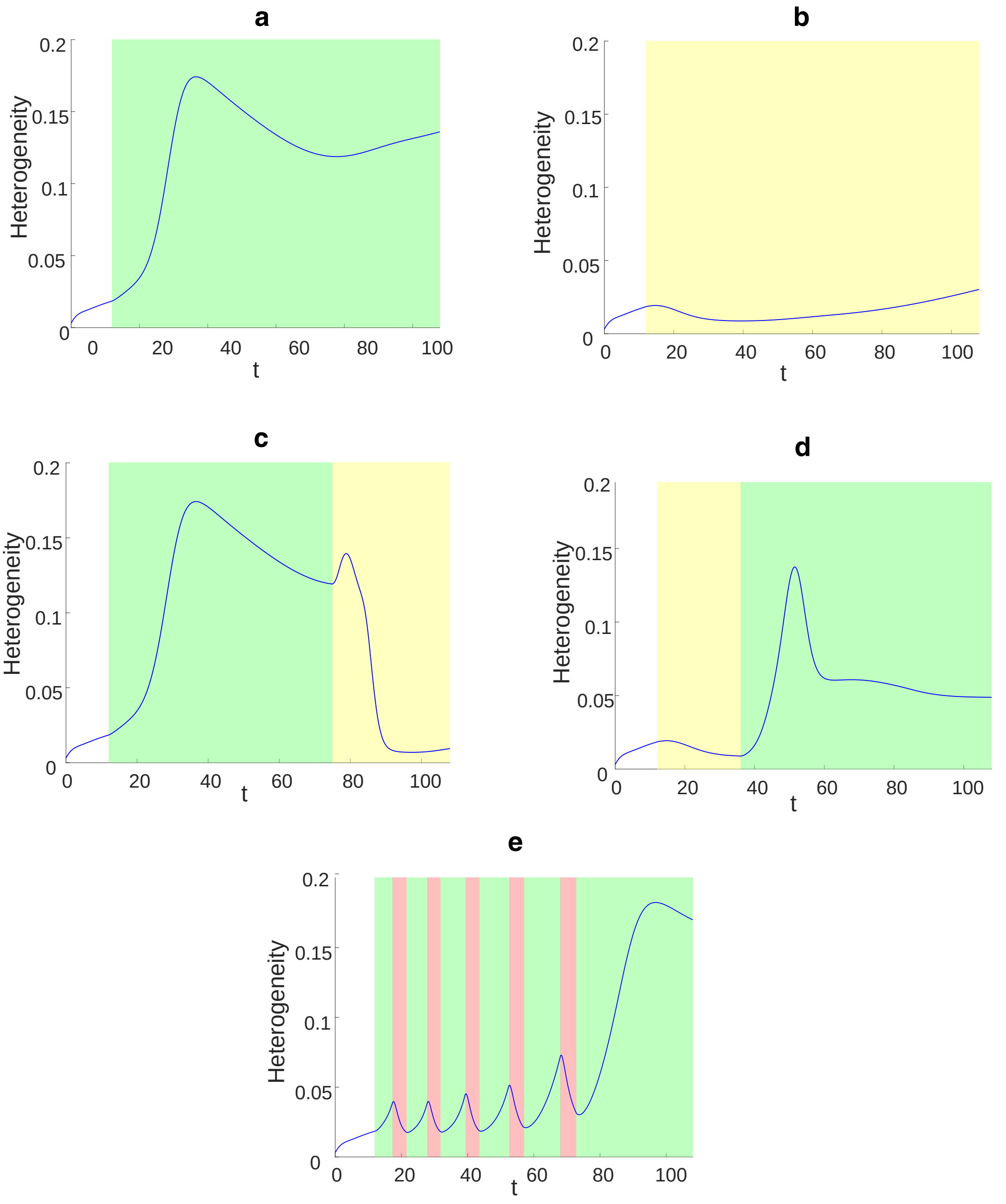


**Figure S1:** Heterogeneity quantification using the sum of variances of structural coordinates for a) continuous treatment with BRAF/MEK inhibitors; b) continuuous HCT treatement; c) combination therapy first BRAF/MEK inhibitors, then HCT; d) combination therapy first HCT, then BRAF/MEK inhibitors; and e) adaptive therapy.

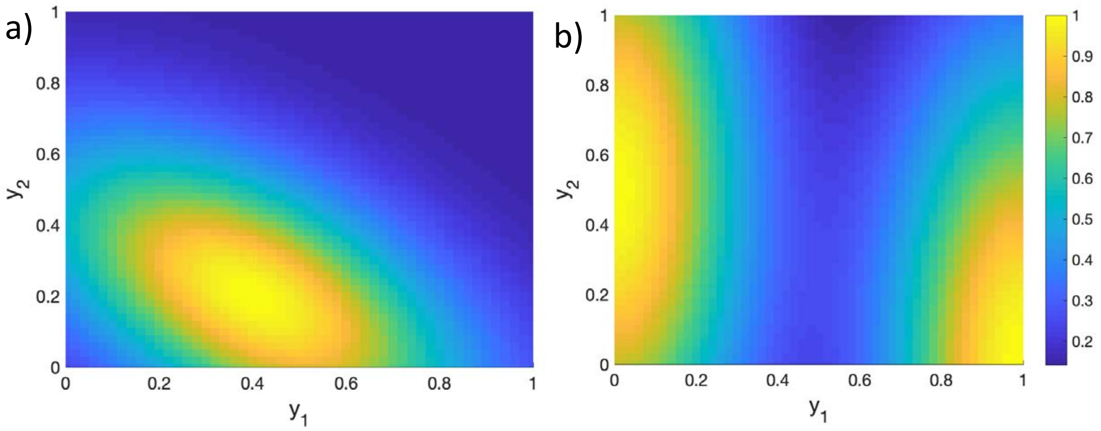


**Figure S2:** Structural drug effectiveness function for BRAF/MEK inhibitor a) and for HCT drug b). The drug acts mainly on cells whose states are located at maxima of the effectiveness function.

## 2.2 Movies

Supplementary Movie 1: Spatial ($x$-) distribution under continuous BRAF/MEK inhibitor treatment. The heatmap represents the normalized cell density in spatial dimensions, averaged over structural dimensions.

Supplementary Movie 2: Structural ($y$-) distribution under continuous BRAF/MEK inhibitor treatment. The heatmap represents the normalized cell density in structural dimensions, averaged over spatial dimensions.

Supplementary Movie 3: Drug induced heterogeneity under continuous BRAF/MEK inhibitor treatment. The false colours of the tumor identify proliferative states (*red*); NCSCs, SMCs, invasive, and pigmented cells (*green*); and URCs (*blue*).

Supplementary Movie 4: Drug induced heterogeneity under continuous HCT treatment. The false colours of the tumor identify proliferative states (*red*); NCSCs, SMCs, invasive, and pigmented cells (*green*); and URCs (*blue*).

Supplementary Movie 5: Drug induced heterogeneity under combination therapy; first BRAF/MEK inhibitors then HCT. The false colours of the tumor identify proliferative states (*red*); NCSCs, SMCs, invasive, and pigmented cells (*green*); and URCs (*blue*).

Supplementary Movie 6: Drug induced heterogeneity under combination therapy; first HCT then BRAF/MEK inhibitors. The false colours of the tumor identify proliferative states (*red*); NCSCs, SMCs, invasive, and pigmented cells (*green*); and URCs (*blue*).

Supplementary Movie 7: Drug induced heterogeneity under adaptive BRAF/MEK inhibitor treatment. The false colours of the tumor identify proliferative states (*red*); NCSCs, SMCs, invasive, and pigmented cells (*green*); and URCs (*blue*).

Supplementary Movie 8: Structural ($y$-) distribution of cancer cells under adaptive BRAF/MEK inhibitor treatment. The heatmap represents the normalized cell density in structural dimensions, averaged over spatial dimensions.
